# Two Novel Lyso-Ornithine Lipids Isolated from an Arctic Marine *Lacinutrix* sp. Bacterium

**DOI:** 10.3390/molecules26175295

**Published:** 2021-08-31

**Authors:** Venke Kristoffersen, Marte Jenssen, Heba Raid Jawad, Johan Isaksson, Espen H. Hansen, Teppo Rämä, Kine Ø. Hansen, Jeanette Hammer Andersen

**Affiliations:** 1 Marbio, Faculty for Fisheries, Biosciences and Economy, UiT-The Arctic University of Norway, Breivika, N-9037 Tromsø, Norway; marte.jenssen@uit.no (M.J.); heba_jr@hotmail.com (H.R.J.); espen.hansen@uit.no (E.H.H.); teppo.rama@uit.no (T.R.); kine.o.hanssen@uit.no (K.Ø.H.); jeanette.h.andersen@uit.no (J.H.A.); 2 Department of Chemistry, Faculty of Natural Sciences, UiT-The Arctic University of Norway, Breivika, N-9037 Tromsø, Norway; johan.isaksson@uit.no

**Keywords:** marine bacteria, lipoamino acid, secondary metabolites, amphiphilic compounds, antibacterial, cytotoxic, anti-cancer

## Abstract

The *Lacinutrix* genus was discovered in 2005 and includes 12 Gram-negative bacterial species. To the best of our knowledge, the secondary metabolite production potential of this genus has not been explored before, and examination of *Lacinutrix* species may reveal novel chemistry. As part of a screening project of Arctic marine bacteria, the *Lacinutrix* sp. strain M09B143 was cultivated, extracted, fractionated and tested for antibacterial and cytotoxic activities. One fraction had antibacterial activity and was subjected to mass spectrometry analysis, which revealed two compounds with elemental composition that did not match any known compounds in databases. This resulted in the identification and isolation of two novel isobranched lyso-ornithine lipids, whose structures were elucidated by mass spectrometry and NMR spectroscopy. Lyso-ornithine lipids consist of a 3-hydroxy fatty acid linked to the alpha amino group of an ornithine amino acid through an amide bond. The fatty acid chains were determined to be iso-C15:0 (**1**) and iso-C16:0 (**2**). Compound **1** was active against the Gram-positive *S. agalactiae*, while **2** showed cytotoxic activity against A2058 human melanoma cells.

## 1. Introduction

Bacteria are the producers of many secondary metabolites that have been developed into drugs, including the tetracycline and aminoglycoside classes of antibiotics [1,2], that has paved the way for better health for millions of people around the world. Most of the bacterial secondary metabolites have been isolated from terrestrial organisms [3], suggesting that the chemical diversity of natural products can be expanded by investigating bacteria from other habitats.

The Arctic marine environment is home to numerous microorganisms thriving in cold water under the stark seasonal changes from midnight sun to polar darkness. Compared to terrestrial microorganisms, the bacteria living under these conditions must be adapted to cold saline water. It is therefore believed that these bacterial species have specialized metabolic systems tailored for survival in this niche environment. Today there are several marketed drugs originating from the marine environment [4]. While most of them were isolated from invertebrates, the true producers of many of these secondary metabolites are now known to be symbiotic bacteria, showing that marine bacteria is a promising source of new bioactive secondary metabolites [5,6]. To increase the likelihood of discovering novel bioactive compounds, one strategy is to search in underexplored places and sources. As the Arctic water is less investigated than warmer waters and terrestrial environments, it represents a potential source for the discovery of novel bioactive bacterial compounds.

The *Lacinutrix* genus belongs to the family *Flavobacteriaceae*, which is the largest family in the Bacteroidetes phylum [7]. The genus consists of Gram-negative marine bacteria that have been isolated from both cold polar waters and warm waters. This genus was first described in 2005 by Bowman and Nichols, when *L. copepodicola* was isolated from an Antarctic marine calanoid copepod [8]. Today the genus includes 12 marine species, five isolated from polar waters and seven from warm waters. In addition to *L. copepodicola*, the polar species includes *L. mariniflava*, *L. algicola* [9] and *L. jangbogonensis* isolated from the Antarctic [10], and *L. himadriensis* isolated from the Arctic [11]. Species isolated from warm waters include *L. iliipiscaria* and *L. gracilariae* isolated from China [12,13,14], *L. cladophorae* and *L. chionocetis* from Japan [13,15], *L. venerupis* from Spain [16] and *L. undariae* and *L. salivirga* isolated from South Korea [17,18]. To date, the studies of *Lacinutrix* sp. have mainly focused on describing novel species; analyzing their genomic and cellular fatty acid content [10,16,19], while their ability to produce secondary metabolites has not yet been assessed.

As part of the current study, two new lyso-ornithine lipids were isolated and characterized. Lyso-ornithine lipids are known to be precursors of ornithine lipids, which are the most common lipoamino acids found in the bacterial membrane. Ornithine lipids are widely distributed in Gram-negative bacteria, but are also present in Gram-positive bacteria. The biosynthesis of ornithine lipids occurs in two steps, where the first step is the formation of lyso-ornithine-lipids from ornithine and 3-hydroxy fatty acyl-acyl carrier protein. Ornithine lipids are formed in the next step by the transfer of an acyl group from fatty acyl-acyl carrier protein to lyso-ornithine [20,21,22].

In the present work, the Arctic marine *Lacinutrix* sp. strain M09B143 was isolated from a *Halichondria* sp. sponge collected in the Barents Sea. The potential of the bacterium to produce bioactive metabolites was evaluated. It was cultivated and the secreted metabolites were extracted from the fermentation broth. The extract was fractionated into six fractions that were tested for antibacterial and cytotoxic activity. Fraction 5 was active against Gram-positive bacteria and was therefore selected for further chemical analysis. This resulted in the isolation and identification of two novel iso-branched lyso-ornithine lipids that were tested for antibacterial and cytotoxic activities.

## 2. Results

### 2.1. Isolation and Identification

**Figure 1:**
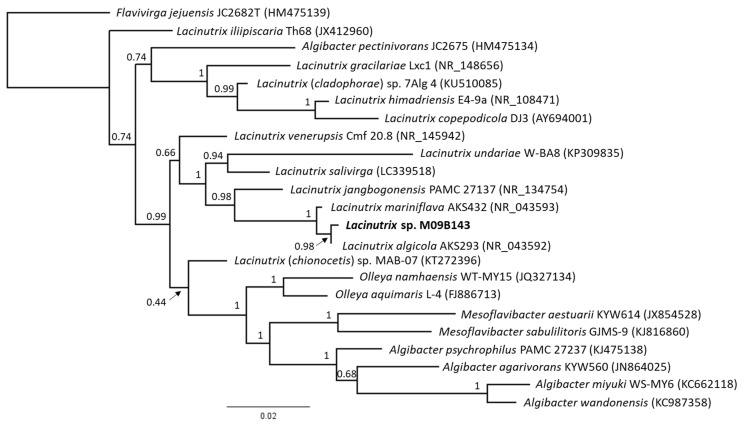
Maximum likelihood tree based on 16S rRNA gene sequences and showing the phylogenetic placement of the strain M09B143 (in bold) within Bacteroidetes. The tree was rooted with *Flavivirga jejuensis* as the outgroup. Branch support is given as aLRT values.

*Lacinutrix* sp. strain M09B143 was isolated from a *Halichondria* sp. sponge collected in the Barents Sea. It was identified as a *Lacinutrix* sp. using 16S rRNA sequencing and Basic Local Alignment Search Tool (BLAST) searches against reference sequences in GenBank. The 16S rRNA gene sequence analysis confirmed that M09B143 was affiliated with the genus *Lacinutrix*, a member of the family *Flavobacteriaceae* and phylum Bacteroidetes, corresponding to the information provided by the Norwegian Marine Biobank Marbank. The bacterium clustered separately on its own branch with *L. algicola* (NR_043592), and sister taxon for this branch was *L. mariniflava* (NR_043592). *L. algicola* and *L. mariniflava* are both isolated from a red alga of the family *Gigartinaceae* [9]. Figure 1 shows the results from the phylogenetic analysis using PhyML. The phylogenetic analysis was also run using the MrBayes 3.2.6 plug-in in Geneious, and the results of this analysis are shown in Supplementary Information Figure S1. There were some differences between the Bayesian Inference tree and the Maximum Likelihood tree, caused by different placement of non-supported nodes in ML and Bayesian analyses and especially the polytomy at one basal node in the tree from MrBayes. The clade consisting of *Lacinutrix* M09B143, *L. algicola*, *L. mariniflava* and *L. jangbogonensis* was statistically supported and topologically similar using both methods.

### 2.2. Bioactivity of Fractionated Extract

**Figure 2:**
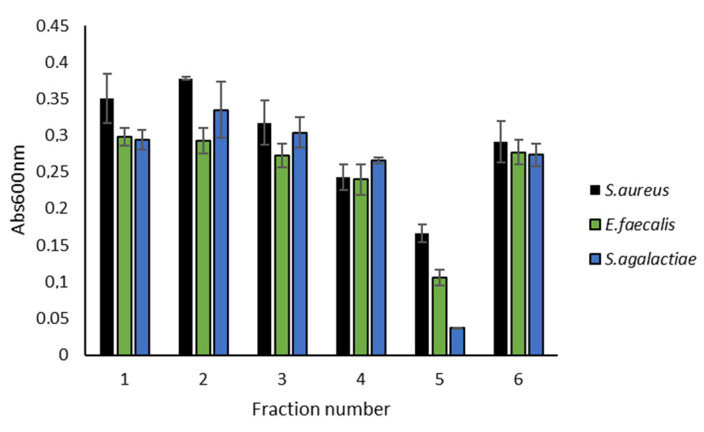
Antibacterial effect of flash fractions 1–6 from M09B143 extract against Gram-positive bacteria tested at 50 µg/mL in a growth inhibition assay (two technical replicates). Fraction 5 was active and was selected for further analysis with UHPLC-HR-MS to identify the compound(s) responsible for the observed activity.

The M09B143 strain was fermented in 2 × 200 mL M19 medium in 1 L flasks. Secondary metabolites excreted into the medium were extracted with Diaion^®^ HP20 resin and eluted with methanol. The bacterial extract was fractionated into six fractions by flash column chromatography and the fractions were tested for antibacterial and cytotoxic activities at 50 µg/mL. Only flash fraction 5, eluting at 100% methanol was active. It was active against the Gram-positive bacteria *Streptococcus agalactiae*, *Enterococcus faecalis* and *Staphylococcus aureus* (Figure 2). The activity appeared to be most potent against *S. agalactiae*, followed by *E. faecalis*. The six fractions were not active against the Gram-negative bacteria *Escherichia coli* and *Pseudomonas aeruginosa*, or against the A2058 human melanoma cells (Figure S2).

### 2.3. Dereplication

Based on the observed antibacterial activity, fraction 5 was subjected to UHPLC-HR-MS analysis. The resulting data were compared to the equivalent data recorded for the inactive fractions 4 and 6 to identify compounds that were exclusively present, or present in higher amounts in fraction 5. The dereplication led to the identification of two compounds, **1**, with elemental composition C_20_H_40_N_2_O_4_ and **2**, with elemental composition C_21_H_42_N_2_O_4_. Compound **1** was the major peak, and **2** was among the most prominent peaks in the MS chromatogram of fraction 5 (Figure S3). Both compounds were only present in very small amounts in the inactive fractions 4 and 6. All other major peaks in the UHPLC-HR-MS chromatogram of fraction 5 were determined to be either media components, or compounds present in comparable amounts in the inactive fractions 4 and 6. Consequently, **1** and **2** were suspected to be responsible for the observed bioactivity of fraction 5. Fragmentation patterns in the UHPLC-HR-MS analysis indicated that they were lipoamino acids, and from their elemental composition and relatively similar retention time, it was assumed that the two compounds differed from each other with a methylene group in the lipid chain. Searches in relevant databases, such as ChemSpider, did not provide any hits that matched the two compounds. Moreover, the dereplication analysis revealed that **1** eluted in three peaks and **2** as two peaks. This indicated that different isomers of both compounds were produced by the bacterium (Figure S4). The three peaks recorded for sample **1** all had the same elemental composition, and the two peaks for sample **2** had the same elemental composition. Fragmentation patterns from MS/MS on the UHPLC-HR-MS were also identical for the different peaks. This strongly indicates that **1** was a mixture of three stereoisomers and that **2** was a mixture of two stereoisomers.

### 2.4. Isolation of Compound **1** and **2**

For purification of the two compounds, upscale cultivation of *Lacinutrix* sp. M09B143 and isolation were performed in two rounds using a preparative HPLC-MS system. The strain was fermented in 64 × 250 mL in round one, which resulted in 25.0 g of dry extract. Fractionation of the extract yielded 515.0 mg of fraction 5. Extensive efforts were put into separating the isomeric variants of each compound from each other. However, due to the lower chromatographic resolution of the preparative column, it was not possible to do so. Therefore, the three variants of **1** were isolated and further processed together, and so were the two variants of **2**. In the text below, compound **1** refers to the sample containing the three variants of **1**, and compound **2** refers to the sample containing the two variants of **2**.

The first isolation step of the two compounds in round one yielded 8.0 mg of **1** and 5.0 mg of **2**. After the second purification step, the yield of **1** was 1.5 mg and 0.6 mg of **2**.

Fermentation and isolation in round two included 56 × 400 mL cultures, which resulted in 28.02 g of dry extract that was fractionated and yielded 1021.2 mg of fraction 5. First purification step of the two compounds with preparative HPLC-MS gave 26.8 mg of **1** and 23.2 mg of **2**. Compound **2** was subjected to a second purification step, resulting in 4.9 mg of **2**.

The two compounds were isolated as light brown waxes; total yield was 28.3 mg of **1** and 5.5 mg of **2**. The purity of the isolated compounds was checked using UHPLC-HR-MS. This revealed that **1** and **2** were completely separated from each other and that the samples only contained minor impurities.

### 2.5. Structure Elucidation

**Figure 3:**
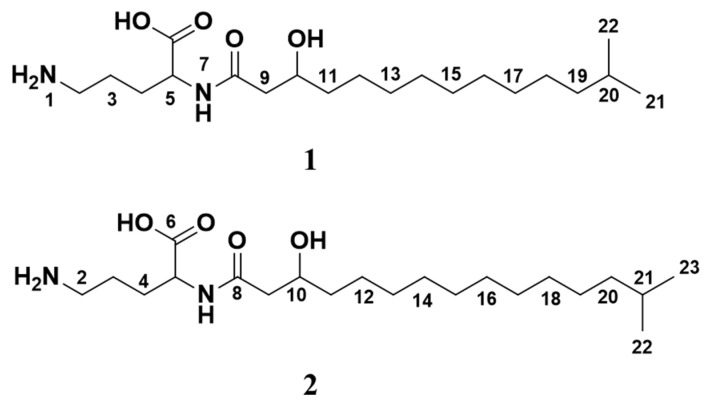
Structures of lyso-ornithine lipids isolated from *Lacinutrix* sp. (**1**): C_20_H_40_N_2_O_4_, (**2**): C_21_H_42_N_2_O_4_.

The structures of **1** and **2** (Figure 3) were elucidated using 1D (^1^H and ^13^C, Table 1) and 2D (HSQC, HMBC, HSQC-TOCSY and COSY, COSY only recorded for **2**). NMR experiments in methanol-*d_3_* and UHPLC-HR-MS analysis. The compounds were determined to consist of a polar ornithine head group linked to a mono-hydroxylated 15:0 (**1**)/16:0 (**2**) iso-fatty acid through an amide bond. The structures of the individual variants of **1** and **2** could not be determined individually, but the presence of two stereoisomers in the 5-position could be observed as two near isochronous C-5 resonances and an unresolvable H-5 multiplet pattern.

**Figure 4:**
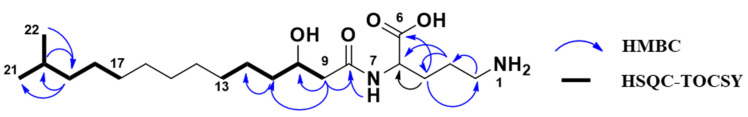
Selected 2D NMR correlations obtained for **1**.

The molecular formula of **1** was calculated to be C_20_H_40_N_2_O_4_ (*m*/*z* 373.3055, [M + H]^+^, calcd 373.3066) by HRESIMS, corresponding to two degrees of unsaturation. The ornithine substructure (atoms 1 to 7) of **1** was assembled through correlations found in the HMBC spectrum (Figure 4 and Figure S5). Deshielding of carbon atom CH_2_-2 (δ_C_ 40.2) places the NH_2_ group at the delta carbon of the amino acid. The carbonyl group was determined to be located at C-6 (δ_C_ 178.0). The fatty acid chain was found to be linked to the polar head group through an amide bond between NH-7 (δ_H_ 7.63) and C-8 (δ_C_ 173.3) based on a HMBC correlation between the two. Furthermore, carbon atoms C-9 to C-13, and C-17 to C-23 were linked through HSQC-TOCSY experiments (Figure 4 and Figure S6), where the C-13 to C-17 resonances overlap in both dimensions. A hydroxy group was placed at carbon atom CH-10 (δ_C_ 69.9) based on HSQC data (Figure S5) and the deshielded shift value of the carbon atom. In agreement with previously reported data for similar compounds [23,24], the central methines (CH_2_-13 to CH_2_-17) could not be individually assigned due to complete signal overlap (Figures S7 and S8). The two equivalent CH_3_ groups (CH_3_-21 and CH_3_-22) of the iso-terminal of the fatty acid were assigned based on ^1^H and HMBC spectrum analysis, and were furthermore linked to a -CH-CH_2_-CH_2_- fragment (CH-20 (δ_C_ 29.0), CH_2_-19 (δ_C_ 40.1) and CH_2_-18 (δ_C_ 28.4)) through HMBC and HSQC-TOCSY correlations. Consequently, the structure of **1** was assigned as 5-amino-2-(3-hydroxy-13-methyltetradecanamido) pentanoic acid.

Through HRESIMS analysis, **2** was determined to have a molecular formula of C_21_H_42_N_2_O_4_ (*m*/*z* 387.3212 [M + H]^+^, calcd 387.3223). The structure of **2** (Figure 3) was assigned by analyzing the data from ^1^H, ^13^C, HSQC, HMBC, HSQC-TOCSY and COSY NMR experiments (Figures S9–S13). The structure of **2** was unambiguously assigned in a similar manner as described above for **1** and was found to have an extension of the fatty acid chain by a CH_2_-group compared to **1** and was consequently assigned as 5-amino-2-(3-hydroxy-14-methylpentadecanamido) pentanoic acid.

### 2.6. Bioactivity Testing of Isolated Compounds

#### 2.6.1. Antibacterial Assay

**Figure 5:**
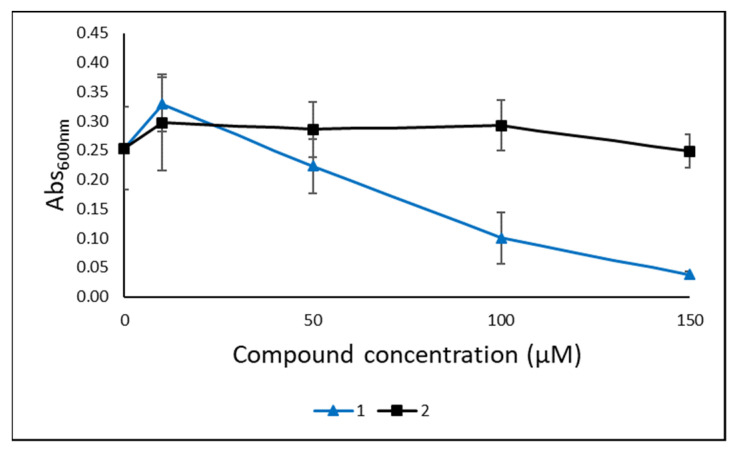
Antibacterial activity of **1** and **2** tested in a growth inhibition assay against the Gram-positive *S. agalactiae*. The assay was performed in three independent experiments, each with three technical replicates.

The two lyso-ornithine lipids were tested for antibacterial activity against the Gram-positive bacteria *S. agalactiae*, *E. faecalis* and *S. aureus*, and against the Gram-negative bacteria *E. coli* and *P. aeruginosa* in a growth inhibition assay in three biological replicates, each containing three technical replicates. The compounds were tested at 10, 50, 100 and 150 µM. As shown in Figure 5, **1** was active against *S. agalactiae*, while **2** showed no activity. A dose-response curve was observed for **1**, with minimum inhibitory concentration between 100 and 150 µM. Compound **1** also had modest effect against *E. faecalis* and *S. aureus* at the highest concentrations, but visible growth was observed in the wells at all concentrations, so complete growth inhibition was not achieved (Figure S14). Neither of the compounds were active against the Gram-negative bacteria (Figure S15).

#### 2.6.2. Cytotoxic Effect of Isolated Lyso-Ornithine Lipids

**Figure 6:**
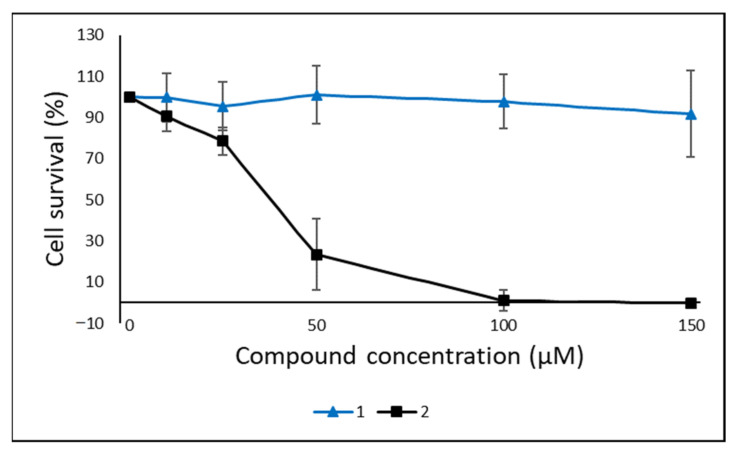
Cytotoxic activity of **1** and **2** against A2058 human melanoma cells. The compounds were tested in three experiments with at least eight technical replicates in total.

The cytotoxicity of the two lyso-ornithine lipids was evaluated against human melanoma cell line A2058 and the non-malignant lung fibroblasts MRC-5 cell line at the concentrations 10, 25, 50, 100 and 150 µM. Some cytotoxic activity against the A2058 cell line was observed for **2**, with 23% cell survival at 50 µM, and ~0% cell survival at 100 and 150 µM (Figure 6). Compound **1** showed no activity against A2058 cells. Neither of the compounds were active against MRC-5 cells (Figure S16). The compounds were tested in three biological replicates with at least eight technical replicates in total.

## 3. Discussion

The antibacterial activity of a fractionated extract from the Arctic marine bacterium *Lacinutrix* sp. led to the identification of two novel lyso-ornithine lipids, **1** and **2**.

Lyso-ornithine lipids are amphiphilic due to their nonpolar fatty acid chain and their polar amino acid head group. Previous studies from our group have identified amphiphilic compounds with antibacterial and cytotoxic activities [23,25]. This includes Lipid 430, with similar structure as the lyso-ornithine lipids. Lipid 430 and **2** have the same iso-branched fatty acid chain, they differ at the head group where Lipid 430 has two serine amino acids whereas **2** has one ornithine amino acid. Lipid 430 was active against the Gram-positive bacterium *S. agalactiae* and against A2058 human melanoma cells. In addition, lipoamino acids are reported to have various bioactivities, such as antibacterial, insecticidal, hemolytic, coagulant and macrophage activity [26,27,28]. Hence, it was likely that the two isolated compounds would be bioactive. After isolation, the two compounds were tested for antibacterial and cytotoxic activities. Compound **1** had some effect against Gram-positive bacteria, particularly *S. agalactiae*, and **2** was moderately cytotoxic to A2058 human melanoma cells. Considering the similarities in the structures of **1** and **2**, this discrepancy in bioactivity was unanticipated. As the compounds are mixtures of isomers, this could be a factor for the discrepancy in activity. However, based on our data, the isomers have the same iso-branched fatty acid linked to an ornithine head group, therefore, the differences in observed bioactivity are most likely due to the different length of the fatty acid chain. The length of the fatty acid chain is known to affect the bioactivity of amphiphilic compounds. For example, Nashida et al. (2018) [29] synthesized mannosylerythritol lipids with various lipid chain length with different antibacterial activity. A study from Tareq et al. (2019) [30] also shows how small differences in the fatty acid chain can affect the bioactivity of amphiphilic compound. They isolated two gageostatins that showed differences in activity against various bacteria and fungi. The only difference between the two isolated gageostatins was a CH_2_ in the lipid chain, similar to the differences between **1** and **2** in the present study.

The two isolated lyso-ornithine lipids showed no activity against the Gram-negative bacteria. This is likely due to the lipopolysaccharide on the outer membrane of Gram-negative bacteria, making it harder for the compounds to access the membrane, as the bioactivity of amphiphilic compounds is commonly due to membrane interactions. Tahara et al. (1977) [31] reported a lyso-ornithine lipid with the same molecular formula as **2**, but with an unbranched fatty acid chain instead of an iso-branched chain, that killed the Gram-negative *E. coli* and *P. aeruginosa* in liquid cultures at 360 µg/mL and 480 µg/mL, respectively. These concentrations are 6-9 times higher than the maximum concentration used in our study, and much higher compared to minimum inhibitory concentrations of marketed antibiotics [32], indicating a fairly weak activity against Gram-negative bacteria.

As lyso-ornithine lipids are precursors for ornithine lipids, it was possible that the extract could contain ornithine lipids. The UHPLC-HR-MS data were therefore specifically checked for the presence of such compounds, but no signals that matched the mass and elemental composition of potential ornithine lipids were detected, indicating that no ornithine lipids were produced. This could be due to the growth conditions used in this study, as the membrane lipid composition can be changed as part of the regulation of membrane fluidity. The amount of iso-branched lipids and lipoamino acids in the membrane is affected by temperature and cultivation conditions [33,34,35,36]. Some bacteria produce lipoamino acids only under limiting phosphate conditions, while others produce them regularly [37,38,39,40].

In the present study we found that lyso-ornithine lipids have some antibacterial and cytotoxic activities. Previous bioactivity studies of lyso-ornithine lipids are limited. In addition to the mentioned study of Tahara et al. (1977), they include a study by Williams et al. (2019) [41], where a lyso-ornithine lipid with good surface activity was described. Surface activity is a feature possessed by surfactants, which are compounds with amphiphilic nature. Biosurfactants (surfactants produced by microorganisms) have the potential to replace chemical surfactants within industrial applications such as remediation of heavy metal and hydrocarbon-contaminated sites, soil washing technology and in cosmetics. In addition, they are known to have various bioactivity properties. These properties include cytotoxicity and antibacterial activity, and are due to their interaction with membranes of target cells, affecting the integrity and stability of the membranes [42,43,44,45]. From this, it is likely that the activity of **1** and **2** is a result of the two compounds interacting with the membranes of the bacteria and the human melanoma cells.

The approach used in this study, investigating underexplored Artic marine bacteria for the production of novel compounds resulted in the characterization of two compounds not described before, showing the potential of Arctic marine bacteria as a source for novel compounds. Bioassay-guided isolation was used to identify the two compounds, as the selection of fractions for further analysis was based on the observed activity in the bioassays. The use of phenotypic bioassays resulted in the isolation of two active compounds with unspecific mode of action. The activity places them outside the potency level needed to be considered relevant for further development toward becoming commercially available pharmaceuticals. Despite of being widely studied, with a few exceptions, the use of biosurfactants within the pharmaceutical industry is today limited. Regarding replacing biosurfactants with chemical surfactants, biosurfactants are today used in cosmetics and in food, but in other industrial applications such as bioremediation and antifouling, the research is still at laboratorial level [46,47]. However, as the research continues, that may change one day.

## 4. Materials and Methods

### 4.1. Sampling and Identification of Lacinutrix sp.

The strain was isolated from a *Halichondria* sp. sponge in the Barents Sea at 74°22′12” N and 19°11′54.2652 E, in January 2009. Glycerol stocks of the bacterium were prepared and provided by Marbank. The bacterial glycerol was plated onto FMAP agar (15 g Difco Marine Broth (Becton Dickinson and Company, Franklin Lakes, NJ, USA), 5 g peptone from casein, enzymatic digest (Sigma, St. Louis, MS, USA), 15 g/L agar, 700 mL ddH_2_O, and 300 mL filtrated sea water), and incubated at 10 °C until sufficient growth. The characterization of the bacterial strains was done by sequencing of the 16S rRNA gene through colony PCR and Sanger sequencing as described previously [48]. The primer set used for gene amplification was the 27F primer (forward primer; 5′-AGAGTTTGATCMTGGCTCAG) and the 1429R primer (reverse primer; 5′-TACCTTGTTACGACTT), both from Sigma. The PCR product was sequenced at the University Hospital of North Norway (Tromsø, Norway). The forward and reverse sequences obtained were assembled using the Geneious Prime^®^ 2021.0.3 software (https://www.geneious.com/) (accessed on 2 July 2021), with the built-in Geneious assembler (sequences trimmed using a 0.05 error probability limit). The *Lacinutrix* M09B143 16S rRNA sequence was deposited in Genbank with the following accession number MZ414169. Reference sequences for the phylogenetic analysis were obtained from Genbank and were selected among top BLAST results of the M09B143 sequence and from recent phylogenetic studies on *Lacinutrix* sp. strains (Supplementary Table S1). The multiple sequence alignment of 23 sequences (including the outgroup *Flavivirga jejuensis*) was conducted using the multiple sequence alignment plug-in Clustal Omega 1.2.2 [49] in Geneious, using the default settings. The alignment was manually adjusted, resulting in a final alignment of 1413 bp length.

Phylogenetic analysis was conducted using the online version of PhyML 3.0 (http://www.atgc-montpellier.fr/phyml/) (accessed on 2 July 2021) [50], and Smart Model Selection [51] was used to select the appropriate substitution model, using the Akaike Information Criterion (AIC) as selection criterion and aBayes for branch support. This suggested the following model to be most appropriate for the dataset: GTR + G + I. The tree was rooted with *F. jejuensis*, branch support is given as aLRT (approximate likelihood ratio test) values. In addition, a phylogenetic analysis was conducted on the same alignment, using the MrBayes 3.2.6 [52] plug-in in Geneious. The analysis was run with the GTR substitution model and rate variation gamma, chain length 1,100,000, subsampling frequency 200 and burn-in length 550,000. The resulting consensus tree was built using default settings.

### 4.2. Fermentation

The M09B143 *Lacinutrix* strain was cultivated in 250 and 400 mL M19 medium in 1 L Erlenmeyer flasks at 10 °C with 140 rpm shaking for 2–3 week until sufficient growth. M19 medium was prepared of 1 L Milli-Q water (Merck Millipore), 20 g D-Mannitol (63560), 20 g Peptone (82303) and 20 g Sea Salt (S9883), all from Sigma-Aldrich. Diaion^®^ HP-20 resin beads (13607, Supelco Analytica) activated in methanol (34860, Sigma-Aldrich) for 20 min and washed with Milli-Q water were added to the cultures to extract compounds secreted into the medium. After 3–4 days the resin was separated from the cultures by filtrating the cultures under vacuum using a mesh cheesecloth (1057, Dansk Hjemmeproduktion, Ejstrupholm, Danmark). Resin collected on the cheesecloth were washed with 100 mL Milli-Q water and compounds adsorbed to the resin was eluted with methanol. The elution was done twice at 140 rpm for 1 h in 150 mL methanol per 40 g resin. The extract was vacuum filtered through Whatman Ø 90 mm No. 3 filter (Whatman plc), dried under reduced pressure at 40 °C and stored at −20 °C.

### 4.3. Flash Fractionation, Bioactivity Testing of Flash Fractions, and Dereplication

Extract of M09B143 was dissolved in 90% methanol before Diaion^®^ HP20 resin was added and the sample was dried under pressure at 40 °C. For each sample, 2 g of extract, 2 g of resin and 8 mL methanol were used. Flash column (Biotage^®^ SNAP Ultra, Biotage, Uppsala, Sweden) was prepared with 6.5 g resin activated in methanol for 20 min before rinsing with Milli-Q water. The resin was loaded in the column and equilibrated with 5% methanol before the extract sample was loaded on top of the column. Fractionation was performed with a Biotage SP4^TM^ system using first a step-wise gradient from 5–100% methanol over 36 min (the steps were 5, 25, 50 and 75% methanol, 6 min each, and 100% methanol for 12 min). Then a gradient with methanol:acetone (34850, Sigma-Aldrich) for 4 min and 100% acetone for 12 min was used. The flow rate was 12 mL/min, resulting in 27 sub fractions with 24 mL in each tube. Sub fraction 1–3, 4–6, 7–9, 10–12, 13–15 and 16–27 were pooled together to a total of six flash fractions and dried under pressure at 40 °C.

### 4.4. Dereplication

The samples were analyzed with ESI+ and ESI- ionization mode on a UPLC-QToF-MS for dereplication. The system (all from Waters) consisted of an Acquity UPLC I-class coupled to a PDA detector and a Vion IMS QToF. An Acquity C18 UPLC column (1.7 µm, 2.1 mm × 100 mm) was used for the separation. Milli-Q water was used for mobile phase A and acetonitrile (HiPerSolv, VWR) for mobile phase B, both containing 0.1% formic acid (*v*/*v*) (33015, Sigma). A 12-min gradient increasing from 10% to 90% acetonitrile with flow rate 0.45 mL/min was used. UNIFI 1.9 (Waters) was used to process the data.

### 4.5. Purification of **1** and **2**

The compounds were purified in two different isolation rounds.

#### 4.5.1. Purification Round One

A preparative HPLC-system (Waters) with a 600 HPLC pump, a 2996 photo diode array detector, a 3100 mass spectrometer and a 2767 sample manager was used to isolate the two compounds. MassLynx version 4.1 was used to control the system. The mobile phases consisted of A; Milli-Q water and B; acetonitrile (Prepsolv^®^, Merck), both containing 0.1% formic acid (*v*/*v*), and flow rate was set to 6 mL/min. Atlantis Prep dC18 column (10 µm, 10 mm × 250 mm) (Waters) was used for the initial separation of the two compounds with gradient 10–88% acetonitrile over 13 min. XSelect CSH Prep Fluoro-Phenyl column (5 µm, 10 mm × 250 mm) (Waters) was used for final purification of **1**, gradient 10–76% acetonitrile over 10 min. For the final purification of **2**, XSelect CSH Phenyl-Hexyl prep column (5 µm, 10 mm × 250 mm) (Waters) was used with gradient 10–54% acetonitrile over 11 min.

#### 4.5.2. Purification Round Two

The initial purification of the compounds in the second round was performed with the same preparative HPLC-system described in the previous section, and the same mobile phases and flow rate. A SunFire C18 OBD column (5 µm, 10 mm × 250 mm) with gradient 50–85% acetonitrile over 10 min was used. A second purification step was performed with **2** on a preparative HPLC-system consisting of Acquity Arc Sample Manager FTN-R, Acquity Arc Quaternary Solvent Manager-R, Acquity Arc Column manager, Acquity QDa Detector and Photodiode Array Detector 2998. Masslynx software was used to control the system. An Atlantis T3, C18 column (3 µm, 3 mm × 150 mm) was used. Flow rate was set to 1.5 mL/min, with gradient 35–55% acetonitrile over 12.5 min.

### 4.6. Antibacterial Activity

Antibacterial activity screening of the fractions and isolated compounds was performed in a growth inhibition assay against the Gram-positive bacteria *S. aureus* (ATCC 25923), *E. faecalis* (ATCC 29122), and *S. agalactiae* (ATCC 12386), and the Gram-negative bacteria *E. coli* (ATCC 259233) and *P. aeruginosa* (ATCC 27853). Flash fractions in the primary screening were dissolved in Milli-Q water with 1% dimethyl sulfoxide (DMSO, D4540, Sigma-Aldrich) to 1 mg/mL, further diluted with Milli-Q water and tested in duplicates at final concentration 50 µg/mL. The isolated compounds were dissolved in DMSO to 20 mM. They were further diluted in Milli-Q water and added to the wells at the final concentrations 10, 50, 100, and 150 µM. The assay was performed as previously described by Kristoffersen et al. (2018) [25]. In total, three biological experiments were performed, with three replicates in each experiment.

### 4.7. Cytotoxic Activity Assay

The cytotoxicity of the fractions in the preliminary screening and of 1 and 2 was tested in an MTS in vitro cell proliferation assay. The fractions and compounds were tested against human melanoma A2058 cells (ATCC, CRL-1147TM). The isolated compounds were in addition tested against normal lung fibroblasts MRC-5 cells (ATCC CCL-171TM). The flash fractions were dissolved in Milli-Q water with 1% DMSO to 1 mg/mL and further diluted in Roswell Park Memorial Institute cell media (FG1383, Merck) with 10% fetal bovine serum (S0115, Biochrom) and tested at 50 µg/mL in three replicates.

Compounds **1** and **2** were dissolved in DMSO to 20 mM, and further diluted in Roswell Park Memorial Institute cell media with 10% fetal bovine serum and tested at the concentrations 10, 25, 50, 100 and 150 µM. One biological experiment with three replicates (test concentration 25 µM was not used here), and two biological experiments with four replicates each were performed. The bioassay was performed as previously described by Kristoffersen et al. (2018) [25].

### 4.8. NMR Spectroscopy

The structures of **1** and **2** were established by 1D and 2D NMR experiments. NMR spectra were acquired in methanol-*d_3_* (CD_3_OH) and 298 K in a 3 mm shigemi tube on a Bruker Avance III HD spectrometer operating at 600 MHz for protons, equipped with an inverse TCI cryo-probe enhanced for ^1^H, ^13^C, and ^2^H.

## 5. Conclusions

*Lacinutrix* sp. was evaluated for its production of bioactive molecules. This resulted in the isolation and characterizing of two novel lyso-ornithine lipids. The bioactive profiling revealed that **1** had some antibacterial activity against the Gram-positive bacterium *S. agalactiae*, with minimum inhibitory concentration between 100 and 150 µM, and that **2** had moderate cytotoxic activity against human melanoma A2058 cells with 23% cell survival at 50 µM, and ~0% cell survival at 100 µM. The length of their lipid chain seemed to affect their activity as (considering the 2-dimentional structure of the two compounds) they only differed with one methylene group in the lipid chain, but showed activity in different bioassays. Should the two compounds be more potent in other bioassays, further studies to determine the structure of the isomers can be performed. This is to our knowledge the first time bioactive molecules have been reported from *Lacinutrix* sp., and the first data describing lyso-ornithine lipids with cytotoxic activity, and with antibacterial activity against Gram-positive bacteria. This shows that exploration of the secondary metabolite content of underexplored bacteria is a viable strategy to discover novel molecules.

## Figures and Tables

**Table 1:** ^1^H and ^13^C assignments for **1** and **2**.

	(1)		(2)	
position	δ_C_, type	δ_H_ (*J* in Hz)	δ_C_, type	δ_H_ (*J* in Hz)
2	40.2, CH_2_	2.95, t (7.3)	40.2, CH_2_	2.95, t (7.3)
3	24.6, CH_2_	1.71, dtd (17.1, 9.5, 8.5, 4.2)	24.6, CH_2_	1.77–1.64, m ^e^
4	30.9, CH_2_	1.91, ddd (10.0, 8.4, 4.8)	30.9, CH_2_	1.90, m
5	54.8, C	4.28, dq (9.9, 3.9, 2.6)	54.8, CH	4.28, d (5.4)
6	178.0, C	-	178.0, C	-
7	-	7.63, d (8.0)	-	7.62, d (8.0)
8	173.7, C	-	173,7, C	-
9a	45.0, CH_2_	2.39, dd (14.3, 3.9)	45.0, CH_2_	2.39, dd (14.4, 4.0)
9b		2.30, dd (14.4, 9.2)		2.30, dd (14.4, 9.2)
10	69.9, CH	3.95, ddt (8.9, 5.8, 3.1)	69.9, CH	3.95, m
11	38.4, CH_2_	1.49, m ^b^	38.4, CH_2_	1.52, m
12	26.6, CH_2_	1.35, m ^c^	26.6, CH_2_	1.48, dq (7.1, 4.4, 3.9)
13	30.7–30.6, CH_2_^a^	1.40–1.22, m ^c^	30.7–30.6, CH_2_^d^	1.40–1.22, m ^f^
14	30.7–30.6, CH_2_^a^	1.40–1.22, m ^c^	30.7–30.6, CH_2_^d^	1.40–1.22, m ^f^
15	30.7–30.6, CH_2_^a^	1.40–1.22, m ^c^	30.7–30.6, CH_2_^d^	1.40–1.22, m ^f^
16	30.7–30.6, CH_2_^a^	1.40–1.22, m ^c^	30.7–30.6, CH_2_^d^	1.40–1.22, m ^f^
17	30.7–30.6, CH_2_^a^	1.40–1.22, m ^c^	30.7–30.6, CH_2_^d^	1.40–1.22, m ^f^
18	28.4, CH_2_	1.40–1.22, m ^c^	30.7–30.6, CH_2_^d^	1.40–1.22, m ^f^
19	40.1, CH_2_	1.16, qd (7.5, 4.2)	28.4, CH_2_	1.40–1.22, m ^f^
20	29.0, CH	1.52, m ^b^	40.1, CH_2_	1.17, q (7.1)
21	22.9, CH_3_	0.86, dd (10.9, 6.7)	29.0, CH	1.77–1.64, m ^e^
22			23.6, CH_3_	0.87, d (6.8)
23	-	-		

^a–f^ Signals are overlapping.

## Data Availability

The data are available within the article and its Supplementary Materials.

## Supplementary Materials

The following are available online, Figure S1: Bayesian Inference tree based on 16S rRNA gene sequence similarity. Figure S2: Fractions 1-6 tested against Gram-negative bacteria and human melanoma A2058 cells. Figure S3: UHPLC-HR-MS base peak intensity chromatogram of flash fraction 5. Figure S4: Extracted UHPLC-HR-MS mass chromatogram of **1** and **2.** Figure S5: HSQC + HMBC (600 MHz, CD_3_OH) spectrum of **1.** Figure S6: HSQC-TOCSY (600 MHz, CD_3_OH) spectrum of **1**. Figure S7: ^1^H NMR (600 MHz, CD_3_OH) spectrum of **1**. Figure S8: ^13^C (151 MHz, CD_3_OH) spectrum of **1**. Figure S9: ^1^H NMR (600 MHz, CD_3_OH) spectrum of **2**. Figure S10: ^13^C (151 MHz, CD_3_OH) spectrum of **2**. Figure S11: HSQC + HMBC (600 MHz, CD_3_OH) spectrum of **2**. Figure S12: HSQC-TOCSY (600 MHz, CD_3_OH) spectrum of **2**. Figure S13: COSY (600 MHz, CD_3_OH) spectrum of **2**. Figure S14: Antibacterial activity of **1** and **2** against Gram-positive bacteria. Figure S15: Antibacterial activity of **1** and **2** against Gram-negative bacteria. Figure S16: Cytotoxic activity of **1** and **2** against non-malignant lung fibroblast cell line MRC-5. Table S1: 16S rRNA sequences used in the phylogenetic analysis of *Lacinutrix* M09B143.

## Sample Availability

Samples of the compounds are not available from the authors.
