# Supplementary material for: Two Novel Lyso-Ornithine Lipids Isolated from an Arctic Marine Lacinutrix sp. Bacterium
Source: Molecules. 2021 Aug 31;26(17):5295. doi: 10.3390/molecules26175295 (PMC8434205; doi:10.3390/molecules26175295)
Supplement: Supplementary file 1 [file molecules-26-05295-s001.zip › molecules-1304561-supplementary.pdf]

Supplementary Materials

Two Novel Lyso-Ornithine Lipids Isolated from an Arctic Marine *Lacinutrix* sp. Bacterium

Venke Kristoffersen [1,*], Marte Jenssen [1], Heba Raid Jawad [1], Johan Isaksson [2], Espen H. Hansen [1], Teppo Rämä [1], Kine Ø. Hansen [1] and Jeanette Hammer Andersen [1]

[1] Marbio, Faculty for Fisheries, Biosciences and Economy, UiT-The Arctic University of Norway, Breivika, N-9037 Tromsø, Norway; marte.jenssen@uit.no (M.J.); heba_jr@hotmail.com (H.R.J.); espen.hansen@uit.no (E.H.H.); teppo.rama@uit.no (T.R.); kine.o.hanssen@uit.no (K.Ø.H); jeanette.h.andersen@uit.no (J.H.A.)

[2] Department of Chemistry, Faculty of Natural Sciences, UiT-The Arctic University of Norway, Breivika, N-9037 Tromsø, Norway; johan.isaksson@uit.no

* Correspondence: venke.kristoffersen@uit.no

Table S1. The names, accession numbers and lengths (bp) of all 16S rRNA sequences used in the phylogenetic analysis of *Lacinutrix* M09B143. All sequences were acquired from Genbank.

Name/Information	Acc.nr	Length (bp)
Algibacter agarivorans strain KYW560 16S ribosomal RNA gene	JN864025	1452
Algibacter miyuki strain WS-MY6 from South Korea 16S ribosomal RNA gene	KC662118	1441
Algibacter pectinivorans strain JC2675 from South Korea 16S ribosomal RNA gene	HM475134	1442
Algibacter psychrophilus strain PAMC 27237 16S ribosomal RNA gene	KJ475138	1510
Algibacter wandonensis 16S ribosomal RNA gene	KC987358	1443
Flavirhabdus (now *Lacinutrix*) *iliipiscaria* strain Th68 16S ribosomal RNA gene	JX412960	1486
Flavivirga jejuensis strain JC2682 from South Korea 16S ribosomal RNA gene, partial sequence (outgroup)	HM475139	1439
Lacinutrix (*chionocetis*) sp. MAB-07 16S ribosomal RNA gene	KT272396	1421
Lacinutrix (*cladophorae*) sp. 7Alg 4 16S ribosomal RNA gene	KU510085	1478
Lacinutrix algicola strain AKS293 16S ribosomal RNA	NR_043592	1496
Lacinutrix copepodicola strain DJ3 16S ribosomal RNA gene	AY694001	1364
Lacinutrix gracilariae strain Lxc1 16S ribosomal RNA	NR_148656	1444
Lacinutrix himadriensis strain E4-9a 16S ribosomal RNA	NR_108471	1488
Lacinutrix jangbogonensis strain PAMC 27137 16S ribosomal RNA	NR_134754	1443
Lacinutrix mariniflava strain AKS432 16S ribosomal RNA	NR_043593	1454
Lacinutrix salivirga gene for 16S ribosomal RNA	LC339518	1460
Lacinutrix sp. strain M09B143 16S ribosomal RNA gene	Must submit	Must submit
Lacinutrix undariae strain W-BA8 16S ribosomal RNA gene	KP309835	1442
Lacinutrix venerupis strain Cmf 20.8 16S ribosomal RNA	NR_145942	1337
Mesoflavibacter aestuarii strain KYW614 16S ribosomal RNA gene	JX854528	1443
Mesoflavibacter sabulilitoris strain GJMS-9 16S ribosomal RNA gene	KJ816860	1446
Olleya aquimaris strain L-4 16S ribosomal RNA gene	FJ886713	1443
Olleya namhaensis strain WT-MY15 16S ribosomal RNA gene	JQ327134	1441

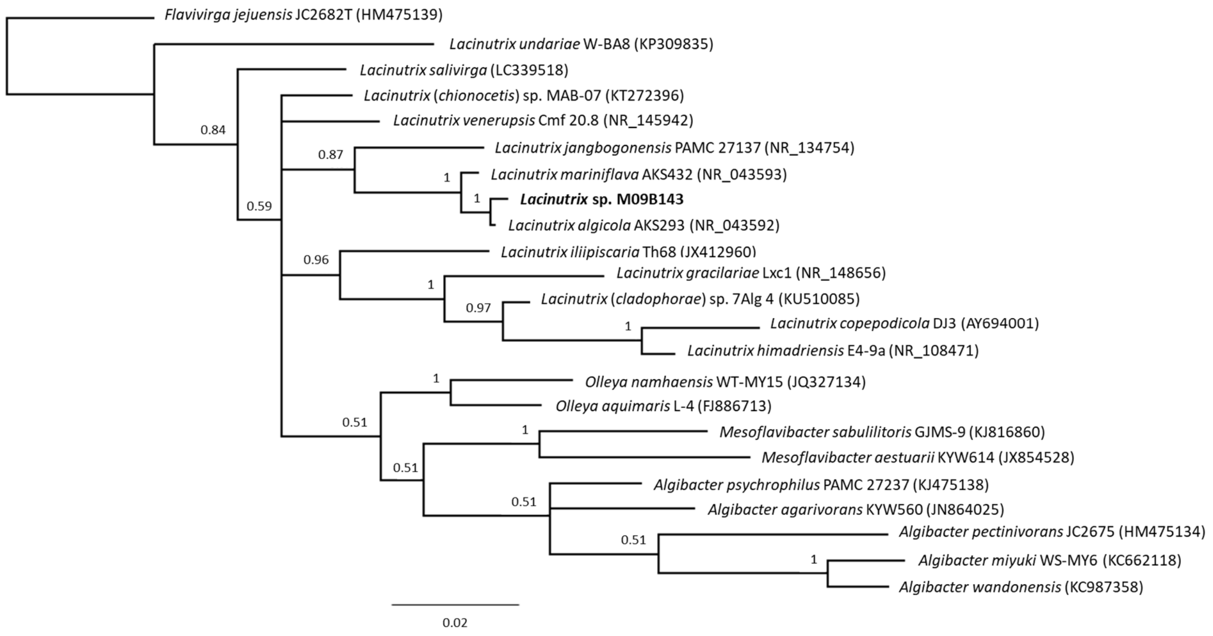

Figure S1. Bayesian Inference tree based on 16S rRNA gene sequence similarity and showing the phylogenetic placement of the isolate M09B143 (in bold) within Bacteroidetes. The tree was rooted with *Flavivirga jejuensis* as the outgroup. Branch support is given Bayesian posterior probability.

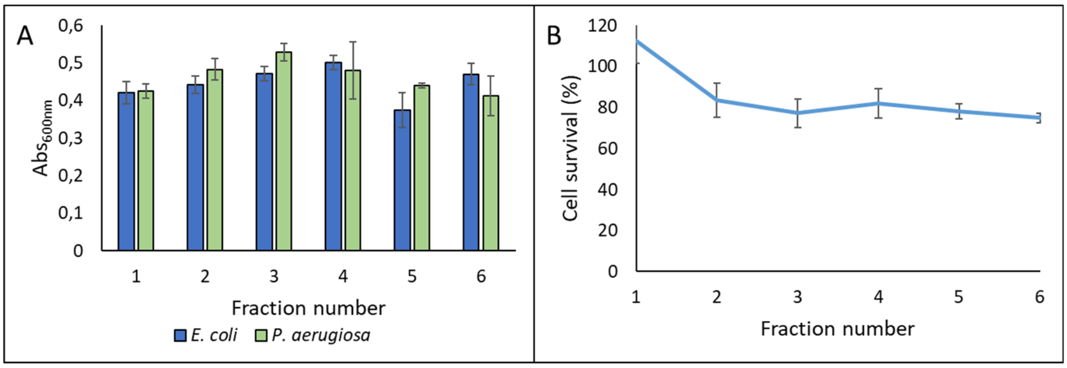

Figure S2. Fractions 1-6 showed no activity against the Gram-negative bacteria *E. coli* and *P. aeruginosa* in the growth inhibition assay, results shown in **A**. There was visible growth in all wells, and the OD values were 0.37 or higher. In comparison, the OD value of fraction 5 was 0.05 when it was active against *S. agalactiae* (Figure 2). The assay was performed in duplicates. Fractions 1-6 showed no activity against human melanoma A2058 cells, results shown in **B**. Cell survival was 75 % or higher for the fractions. The assay was performed with three technical replicates.

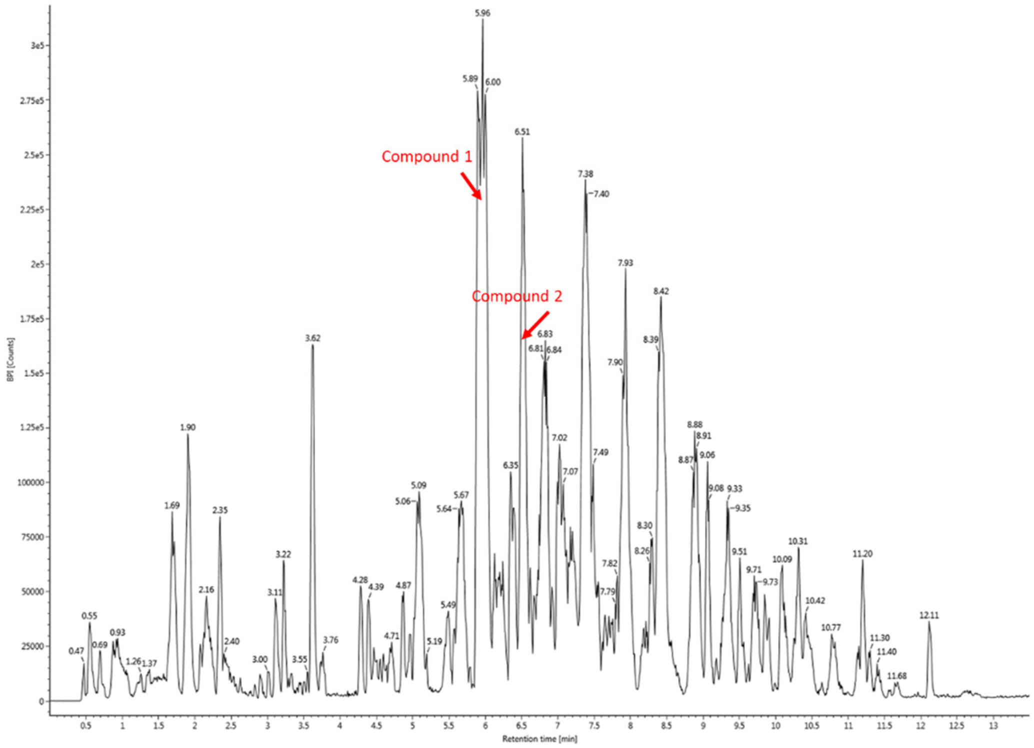

Figure S3. UHPLC-HR-MS base peak intensity chromatogram of fraction 5 of *Lacinutrix* sp., where **1** is the major peak, and **2** among the highest peaks.

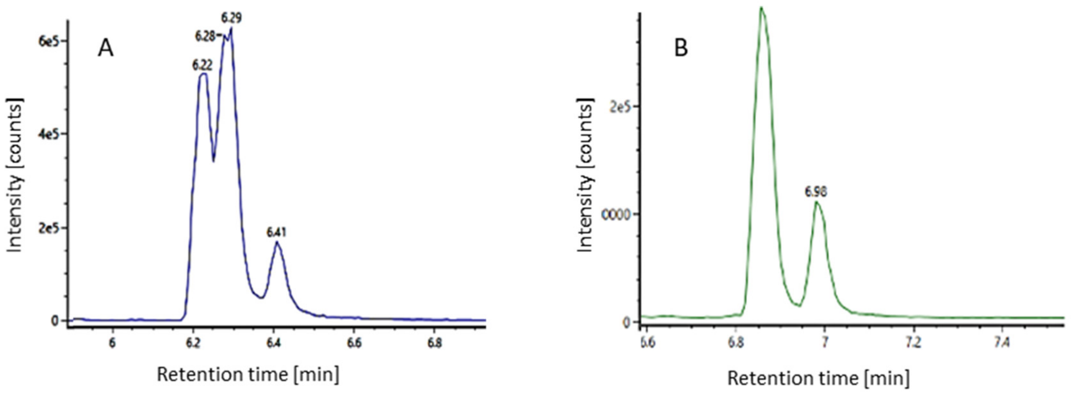

Figure S4. Extracted UHPLC-HR-MS mass chromatogram of **1** shown in **A**, and **2** shown in **B**. Possible isomers were observed, as **1** eluted in three peaks and **2** eluted in two peaks.

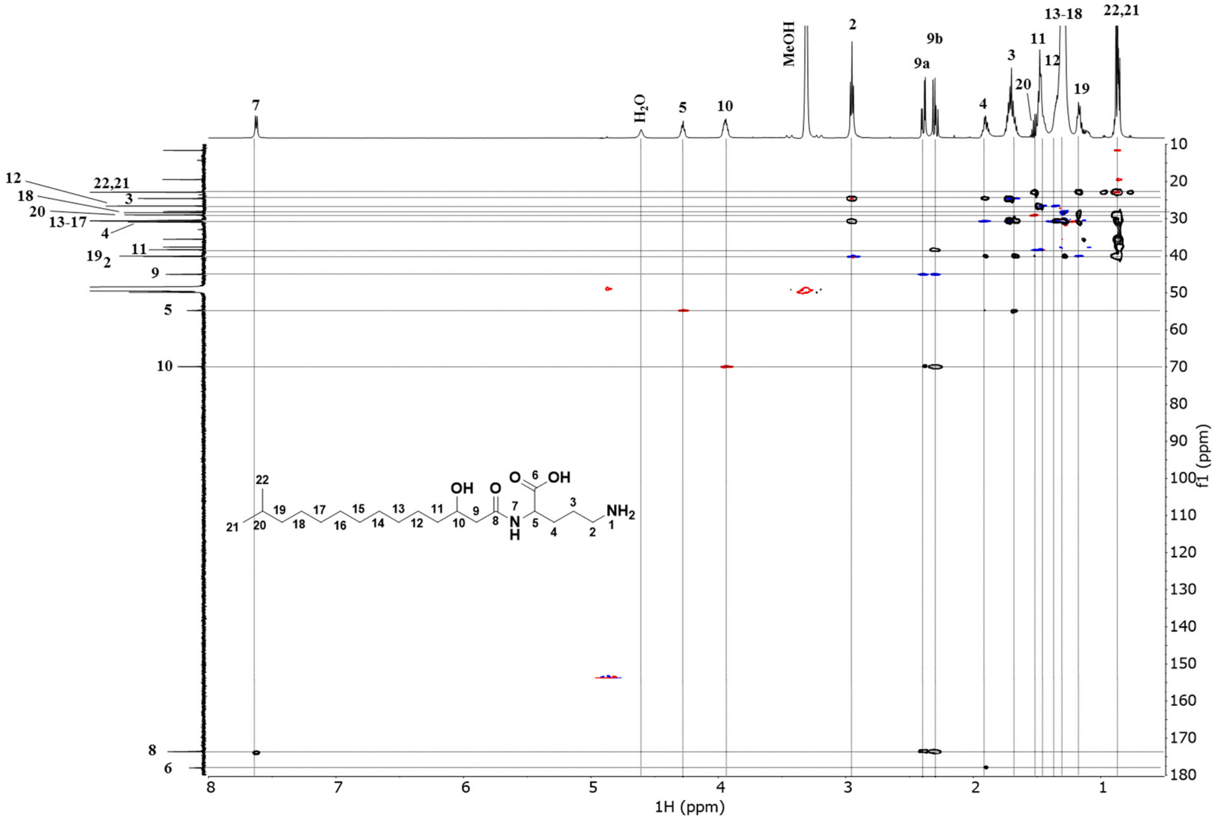

Figure S5. HSQC + HMBC (600 MHz, CD_3OH) spectrum of **1**.

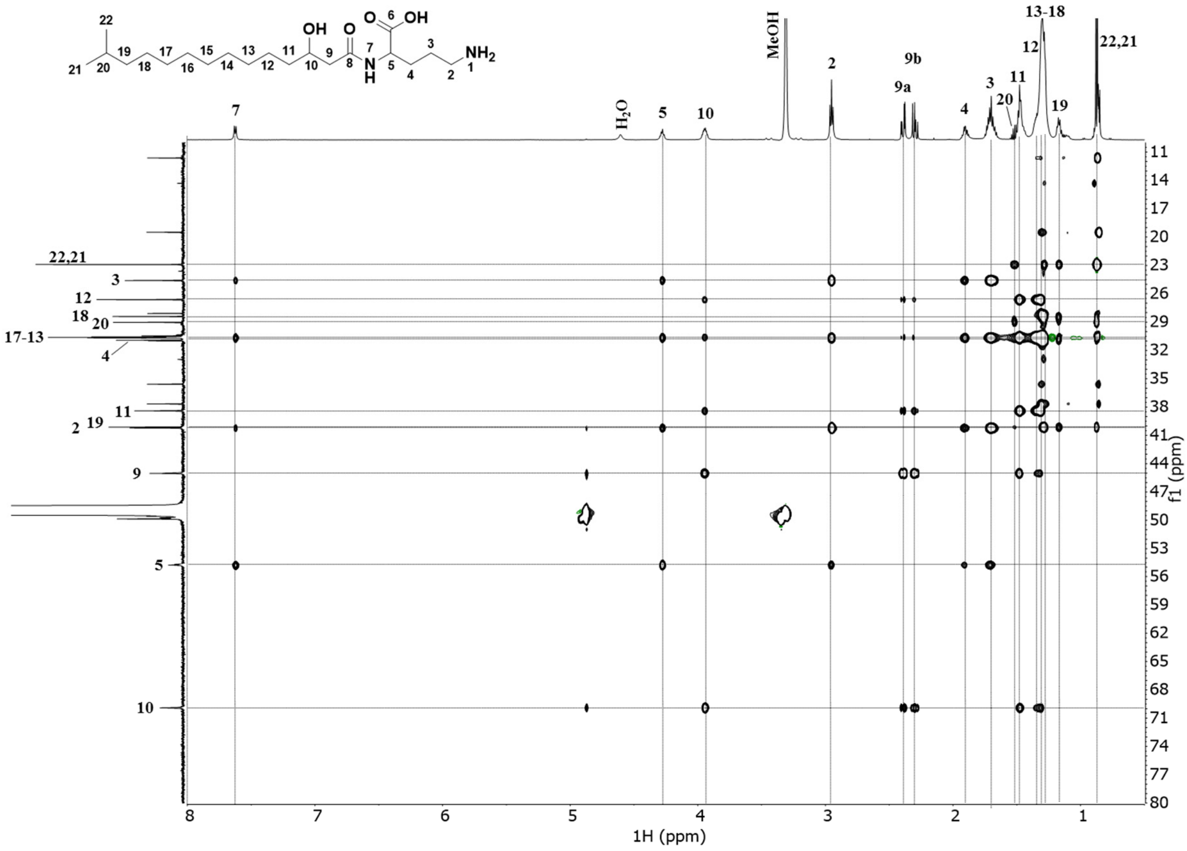

Figure S6. HSQC-TOCSY (600 MHz, CD_3OH) spectrum of **1**.

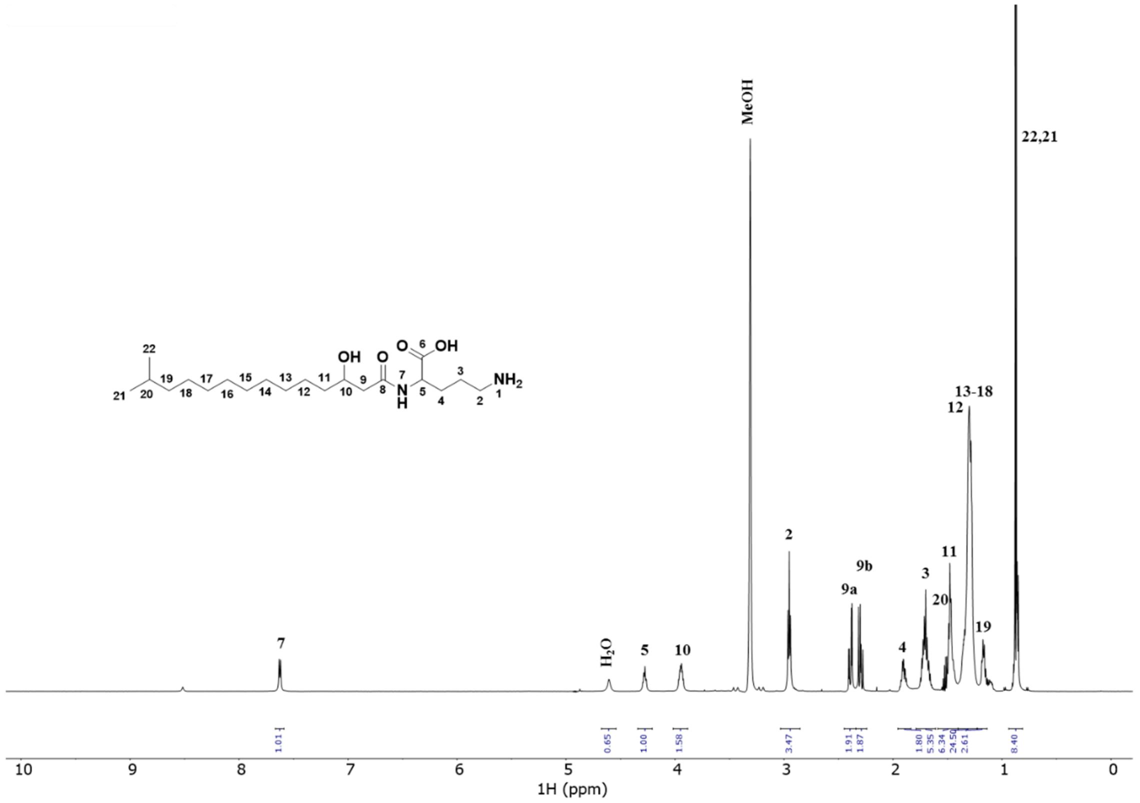

Figure S7. ^{1}H NMR (600 MHz, CD_3OH) spectrum of **1**.

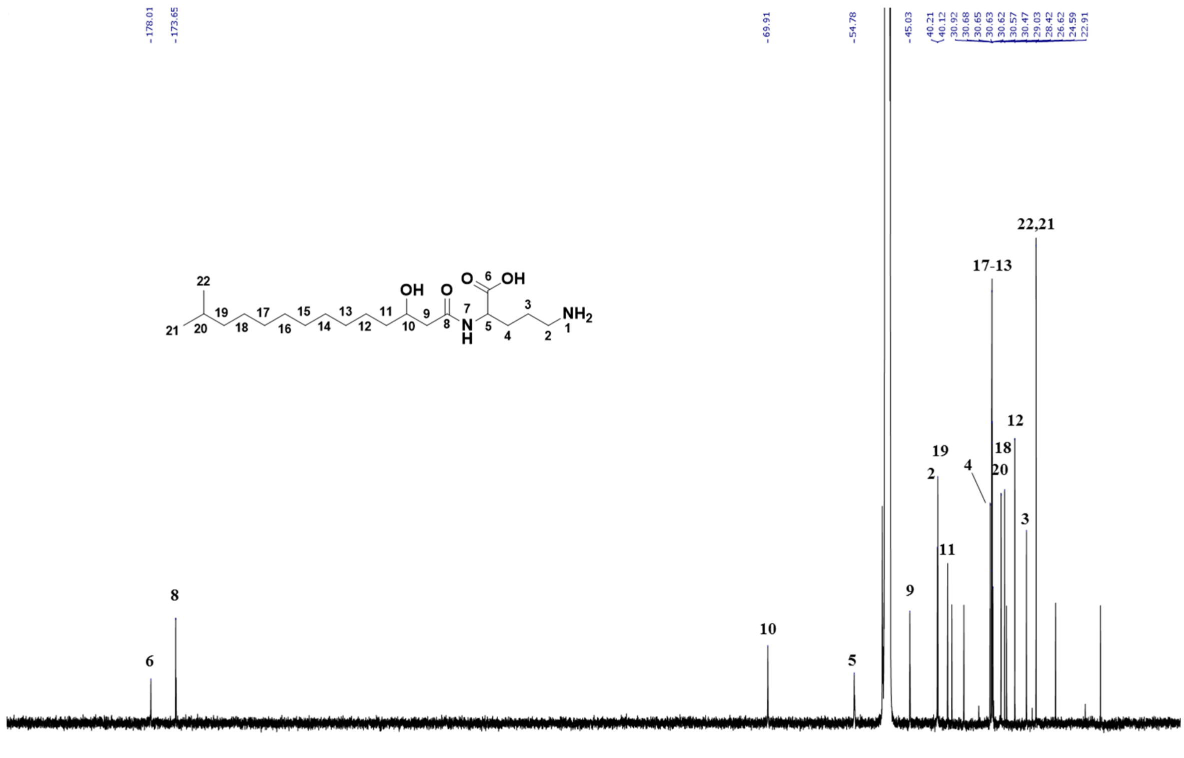

Figure S8. ^{13}C (151 MHz, CD_3OH) spectrum of **1**.

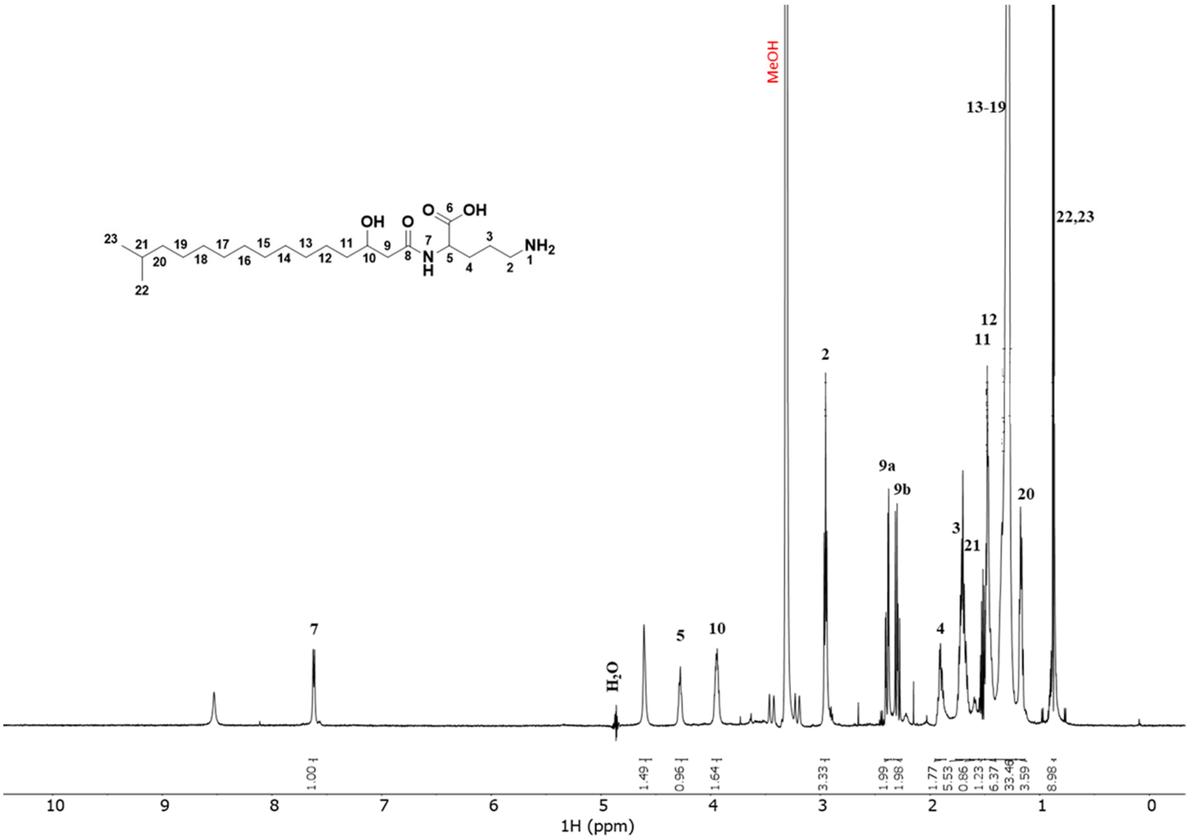

Figure S9. ^{1}H NMR (600 MHz, CD_3OH) spectrum of **2**.

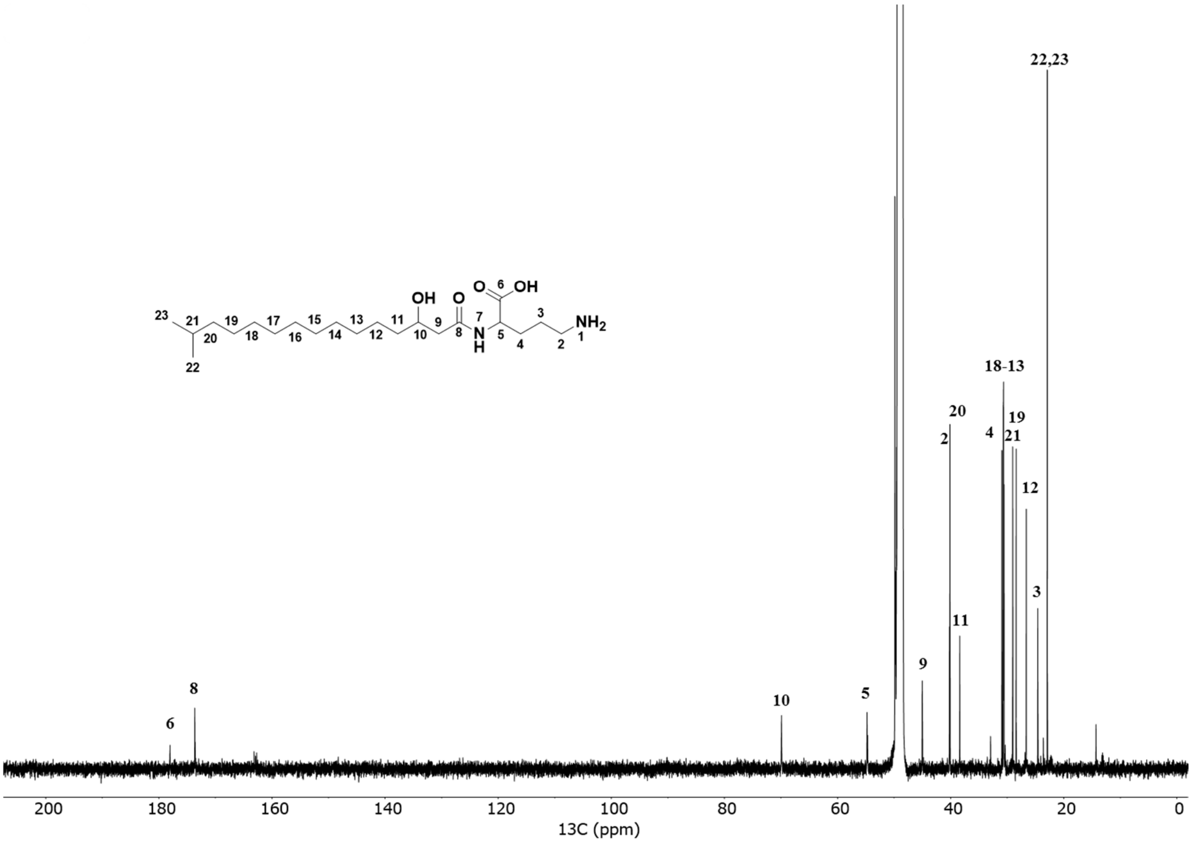

Figure S10. ^{13}C (151 MHz, CD_3OH) spectrum of **2**.

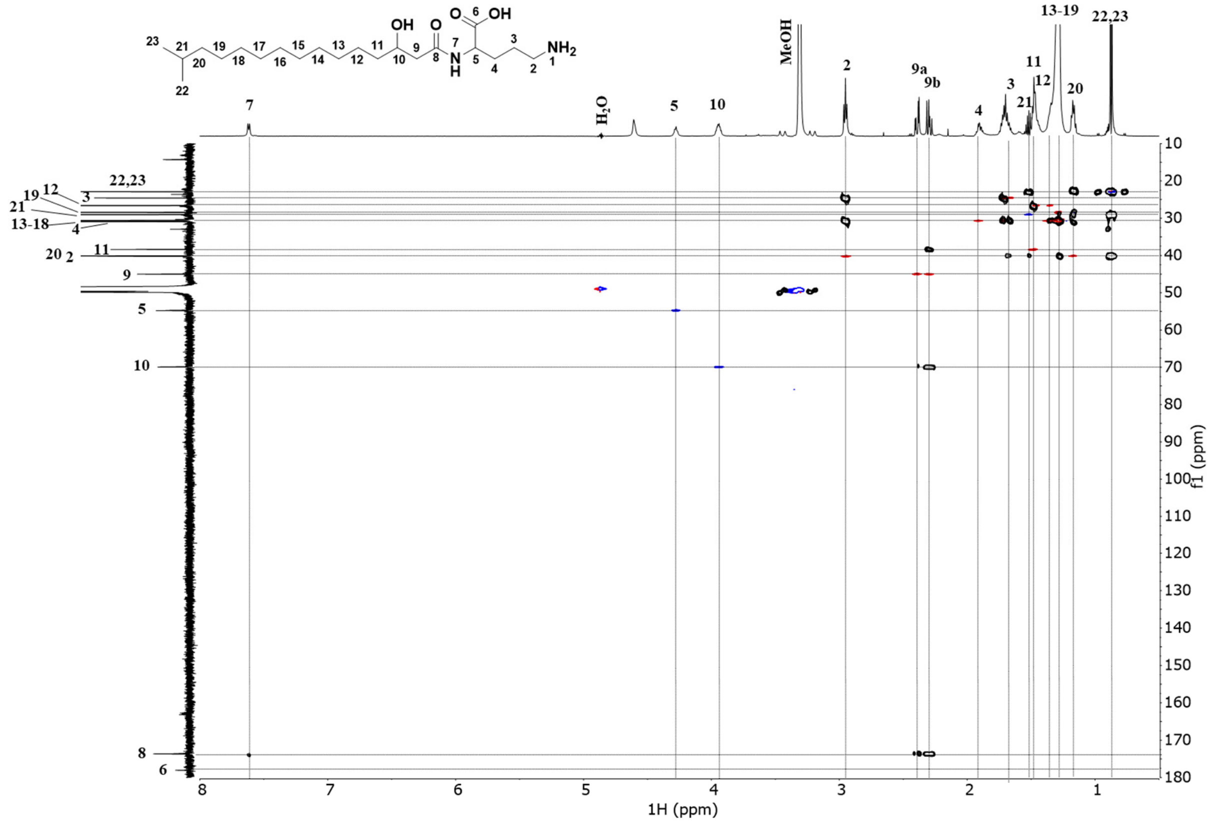

Figure S11. HSQC + HMBC (600 MHz, CD_3OH) spectrum of **2**.

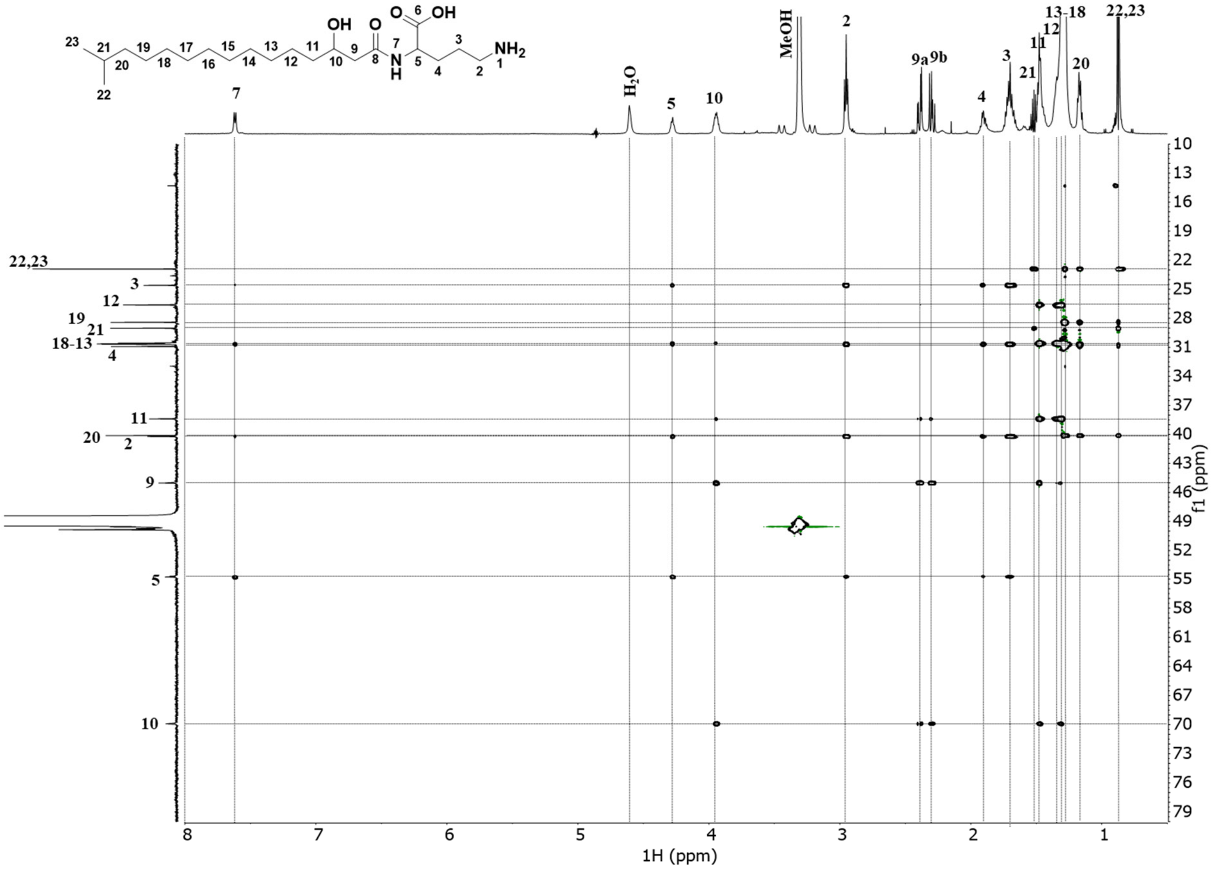

Figure S12. HSQC-TOCSY (600 MHz, CD_3OH) spectrum of **2**.

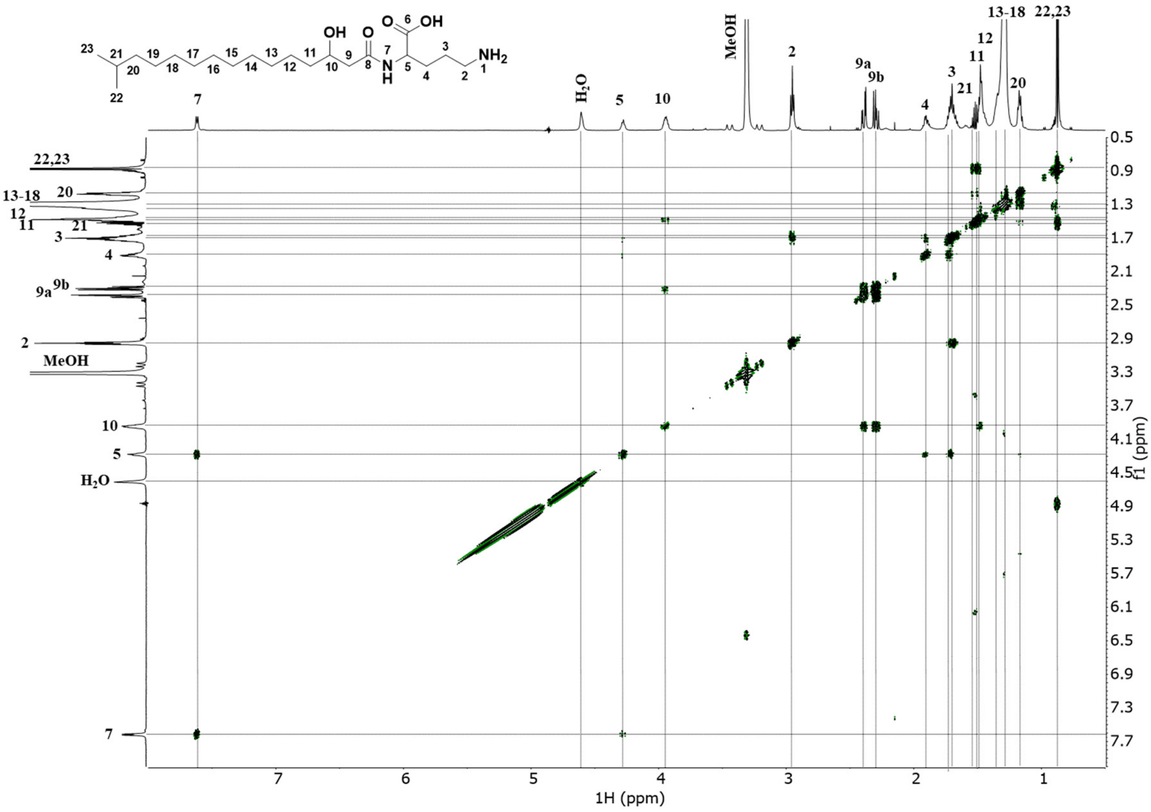

Figure S13. COSY (600 MHz, CD_3OH) spectrum of **2.**

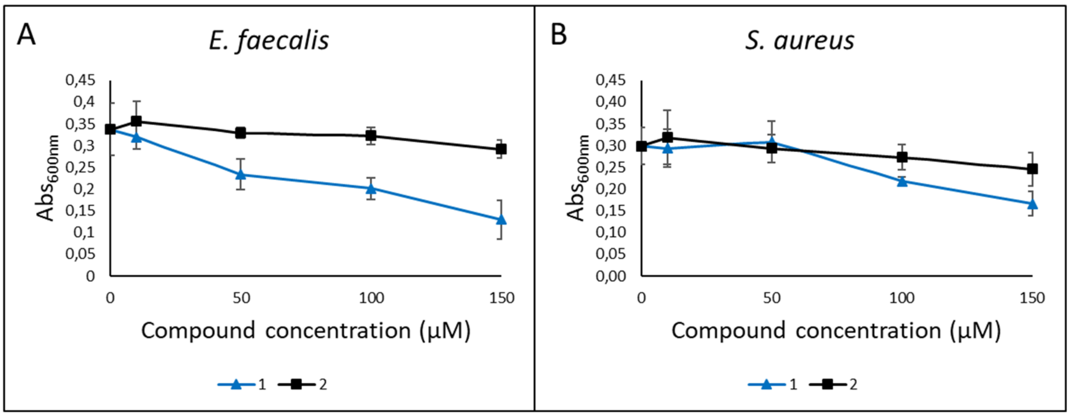

Figure S14. Antibacterial activity of **1** and **2** was tested in a growth inhibition assay. The results are shown for the Gram-positive bacteria *E. faecalis* in A and *S. aureus* in B. The assay was performed in three biological experiments with three technical replicates each.

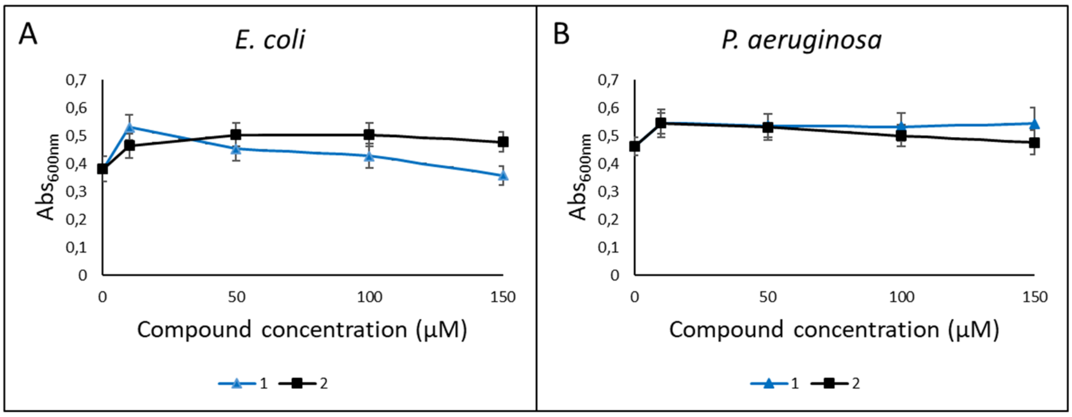

Figure S15. Antibacterial activity of **1** and **2** was tested in a growth inhibition assay. The results are shown for the Gram-negative bacteria *E. coli* in A and *P. aeruginosa* in B. The assay was performed in three biological experiments with three technical replicates each.

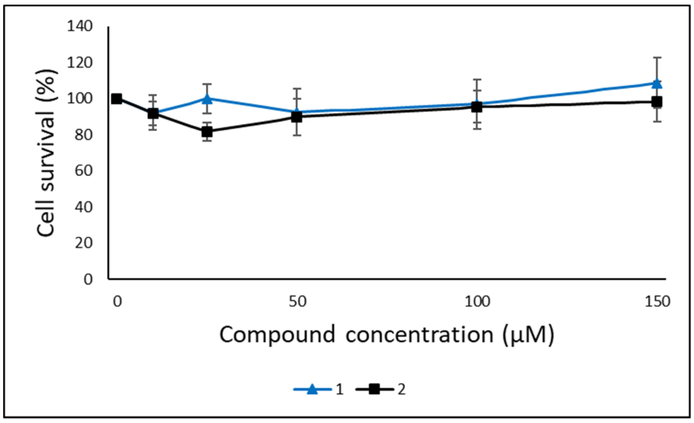

Figure S16. Cytotoxic activity of **1** and **2** was tested against non-malignant lung fibroblasts MRC-5 cells in a viability assay at 10, 25, 50, 100 and 150µM. Three experiments were conducted, one with three replicates (test concentration 25 µM was not used in this setup) and two with four replicates.
